# Supplementary material for: Fatty Acid and Stable Carbon Isotope Composition of Slovenian Milk: Year, Season, and Regional Variability
Source: Molecules. 2020 Jun 23;25(12):2892. doi: 10.3390/molecules25122892 (PMC7356875; doi:10.3390/molecules25122892)
Supplement: Supplementary file 1 [file molecules-25-02892-s001.pdf]

Table S1: δ13C of fatty acids in milk from different geographical location in Slovenia during summer/winter in 2013 and 2014.

| FA (δ13C ‰) | Year | Season | ALPINE Mean ± SD | DINARIC | PANNONIAN | MEDITERRANEAN |
|---|---|---|---|---|---|---|
| C10:0 | 2013 | summer | −26.7 ± 1.4 | −27.3 ± 1.1 | −24.3 ± 1.8 | −28.6 ± 2.1 |
| | 2013 | winter | −22.7 ± 1.4 | −22.7 ± 1.5 | −21.1 ± 0.7 | −24.1 ± 2.3 |
| | **2013** | **average** | **−24.5 ± 2.4** | **−24.3 ± 2.5** | **−22.4 ± 2.0** | **−26.3 ± 3.2** |
| | 2014 | summer | −24.4 ± 1.3 | −23.6 ± 1.9 | −22.7 ± 1.6 | −26.5 ± 0.7 |
| | 2014 | winter | −20.5 ± 1.6 | −19.9 ± 1.5 | −19.7 ± 1.0 | −25.6 ± 0.8 |
| | **2014** | **average** | **−22.5 ± 2.5** | **−21.6 ± 2.5** | **−21.3 ± 2.0** | **−26.1 ± 1.0** |
| C12:0 | 2013 | summer | −28.0 ± 1.3 | −28.5 ± 1.1 | −25.5 ± 1.7 | −30.2 ± 2.0 |
| | 2013 | winter | −23.2 ± 1.3 | −22.9 ± 1.7 | −20.9 ± 0.9 | −25.1 ± 2.9 |
| | **2013** | **average** | **−25.4 ± 2.7** | **−24.9 ± 3.0** | **−22.8 ± 2.6** | **−27.6 ± 3.6** |
| | 2014 | summer | −25.3 ± 1.3 | −24.5 ± 1.9 | −23.1 ± 0.9 | −27.5 ± 0.9 |
| | 2014 | winter | −21.1 ± 1.7 | −20.8 ± 1.8 | −20.4 ± 1.1 | −26.8 ± 0.8 |
| | **2014** | **average** | **−23.2 ± 2.6** | **−22.5 ± 2.6** | **−21.8 ± 1.7** | **−27.2 ± 1.0** |
| C14:1 | 2013 | summer | −29.2 ± 2.5 | −30.9 ± 2.7 | −28.1 ± 3.8 | −32.8 ± 2.9 |
| | 2013 | winter | −26.0 ± 1.7 | −26.6 ± 1.7 | −24.7 ± 0.9 | −27.9 ± 2.5 |
| | **2013** | **average** | **−27.4 ± 2.6** | **−28.1 ± 2.9** | **−26.1 ± 3.0** | **−30.4 ± 3.7** |
| | 2014 | summer | −27.7 ± 1.4 | −26.7 ± 2.9 | −25.9 ± 1.6 | −30.1 ± 1.1 |
| | 2014 | winter | −22.5 ± 1.8 | −22.2 ± 1.1 | −22.3 ± 1.8 | −27.9 ± 1.1 |
| | **2014** | **average** | **−25.1 ± 3.1** | **−24.4 ± 3.1** | **−24.2 ± 2.4** | **−29.0 ± 1.7** |
| C14:0 | 2013 | summer | −26.1 ± 2.5 | −26.6 ± 2.1 | −23.7 ± 1.6 | −29.8 ± 2.4 |
| | 2013 | winter | −22.4 ± 1.3 | −21.9 ± 1.7 | −20.7 ± 1.3 | −23.7 ± 3.0 |
| | **2013** | **average** | **−24.2 ± 2.7** | **−23.6 ± 2.9** | **−22.0 ± 2.0** | **−26.8 ± 4.1** |
| | 2014 | summer | −23.7 ± 1.1 | −23.3 ± 1.8 | −22.5 ± 2.9 | −25.8 ± 0.7 |
| | 2014 | winter | −20.9 ± 1.8 | −19.6 ± 1.4 | −19.9 ± 0.8 | −25.7 ± 0.8 |
| | **2014** | **average** | **−22.3 ± 2.1** | **−21.3 ± 2.4** | **−21.3 ± 2.5** | **−25.7 ± 0.8** |
| C15:0 | 2013 | summer | −35.1 ± 2.2 | −36.1 ± 2.7 | −33.4 ± 3.7 | −38.0 ± 3.0 |
| | 2013 | winter | −30.1 ± 1.5 | −30.7 ± 2.0 | −28.6 ± 1.0 | −31.9 ± 2.0 |
| | **2013** | **average** | **−32.7 ± 3.1** | **−32.8 ± 3.4** | **−30.6 ± 3.5** | **−34.9 ± 4.0** |
| | 2014 | summer | −31.6 ± 1.2 | −30.9 ± 2.1 | −29.7 ± 2 | −33.1 ± 1.0 |
| | 2014 | winter | −26.7 ± 2.1 | −25.8 ± 1.2 | −26.2 ± 1.4 | −31.8 ± 0.8 |
| | **2014** | **average** | **−29.2 ± 3.1** | **−28.2 ± 3.1** | **−28.2 ± 2.5** | **−32.4 ± 1.2** |
| C16:1 | 2013 | summer | −31.0 ± 1.9 | −32.0 ± 2.7 | −30.1 ± 3.6 | −35.0 ± 3.8 |
| | 2013 | winter | −27.0 ± 1.4 | −27.4 ± 1.7 | −25.0 ± 0.9 | −28.6 ± 2.7 |
| | **2013** | **average** | **−28.8 ± 2.6** | **−29.0 ± 3.0** | **−27.1 ± 3.5** | **−31.8 ± 4.6** |
| | 2014 | summer | −29.5 ± 1.7 | −28.2 ± 2.0 | −26.3 ± 1.7 | −31.0 ± 2.0 |
| | 2014 | winter | −23.6 ± 1.7 | −23.3 ± 1.3 | −23.5 ± 0.9 | −29.1 ± 0.8 |
| | **2014** | **average** | **−26.6 ± 3.5** | **−25.6 ± 3.0** | **−25.0 ± 1.9** | **−30.0 ± 2.0** |
| C16:0 | 2013 | summer | −27.3 ± 1.9 | −27.4 ± 0.9 | −22.5 ± 1.7 | −28.2 ± 2.4 |
| | 2013 | winter | −22.9 ± 1.1 | −22.5 ± 1.8 | −21.0 ± 1.0 | −22.5 ± 2.7 |
| | **2013** | **average** | **−24.8 ± 2.6** | **−24.2 ± 2.8** | **−21.6 ± 1.5** | **−25.3 ± 3.8** |
| | 2014 | summer | −24.7 ± 1.6 | −23.7 ± 2.1 | −22.4 ± 1.3 | −27.0 ± 1.2 |
| | 2014 | winter | −22.0 ± 1.8 | −20.8 ± 2.0 | −20.3 ± 1.2 | −26.7 ± 0.5 |
| | **2014** | **average** | **−23.4 ± 2.2** | **−22.1 ± 2.5** | **−21.4 ± 1.6** | **−26.8 ± 1.1** |
| C18:1ω9c | 2013 | summer | −29.8 ± 1.8 | −29.7 ± 2.3 | −26.3 ± 1.5 | −32.1 ± 2.7 |
| | 2013 | winter | −27.3 ± 1.5 | −26.2 ± 1.4 | −25.1 ± 1.0 | −26.4 ± 1.5 |
| | **2013** | **average** | **−28.4 ± 2.1** | **−27.4 ± 2.4** | **−25.6 ± 1.3** | **−29.8 ± 3.7** |
| | 2014 | summer | −27.3 ± 1.3 | −26.7 ± 1.0 | −25.3 ± 1.4 | −28.9 ± 1.5 |
| | 2014 | winter | −25.6 ± 1.7 | −24.6 ± 1.8 | −23.9 ± 1.1 | −31.1 ± 2.3 |
| | **2014** | **average** | **−26.4 ± 1.8** | **−25.6 ± 1.8** | **−24.7 ± 1.4** | **−30.0 ± 2.4** |
| C18:0 | 2013 | summer | −30.8 ± 2.7 | −30.9 ± 2.8 | −28.4 ± 1.1 | −33.8 ± 3.1 |
| | 2013 | winter | −26.8 ± 1.9 | −26.6 ± 1.7 | −24.8 ± 1.3 | −26.8 ± 2.8 |
| | **2013** | **average** | **−28.6 ± 3.0** | **−28.1 ± 3.0** | **−26.3 ± 2.1** | **−30.3 ± 4.6** |

| FA ($\delta^{13}C$ ‰) | Year | Season | ALPINE Mean ± SD | DINARIC | PANNONIAN | MEDITERRANEAN |
|---|---|---|---|---|---|---|
| | 2014 | summer | −28.5 ± 1.3 | −28.1 ± 1.4 | −27.0 ± 1.7 | −31.7 ± 3.9 |
| | 2014 | winter | −25.5 ± 2.0 | −25.0 ± 2.7 | −23.8 ± 0.9 | −30.6 ± 1.8 |
| | **2014** | **average** | **−27.0 ± 2.3** | **−26.5 ± 2.7** | **−25.5 ± 2.1** | **−31.2 ± 3.4** |
| **Bulk** | 2013 | summer | −22.9 ± 1.0 | −23.3 ± 1.7 | −20.7 ± 1.0 | −24.9 ± 1.9 |
| | 2013 | winter | −21.2 ± 1.1 | −21 ± 1.5 | −19.6 ± 0.6 | −22.9 ± 2.2 |
| | **2013** | **average** | **−21.9 ± 1.4** | **−21.8 ± 1.9** | **−20.1 ± 1.0** | **−23.9 ± 2.3** |
| | 2014 | summer | −23.7 ± 1.3 | −22.4 ± 1.7 | −21.2 ± 1.1 | −25.2 ± 1.5 |
| | 2014 | winter | −20.8 ± 1.5 | −20.2 ± 0.9 | −19.2 ± 0.9 | −24.9 ± 1.2 |
| | **2014** | **average** | **−22.3 ± 2.0** | **−21.3 ± 1.7** | **−20.2 ± 1.4** | **−25.1 ± 1.5** |

Table S2: Geographical information of the sampling location

| Sample location | Geographical origin | Latitude | Longitude | Distance near the coast (~m) | Altitude (~m.a.s.l.) |
|---|---|---|---|---|---|
| Brkini | Mediterranean | 45°34'17.8"N | 14°02'35.8"E | 20,000 | 588 |
| Kozina | Mediterranean | 45°36'30.0"N | 13°55'43.9"E | 11,000 | 493 |
| Sežana | Mediterranean | 45°42'09.1"N | 13°51'06.5"E | 10,000 | 360 |
| Maribor | Pannonian | 46°32'50.5"N | 15°38'42.8"E | 176,000 | 162 |
| Korena | Pannonian | 46°31'27.1"N | 15°46'20.1"E | 188,000 | 350 |
| Ptuj | Pannonian | 46°25'18.3"N | 15°52'05.4"E | 186,000 | 232 |
| Rogaška | Pannonian | 46°14'09.8"N | 15°38'08.9"E | 159,000 | 224 |
| Konjice | Pannonian | 46°20'16.1"N | 15°25'23.6"E | 152,000 | 322 |
| Šentilj | Pannonian | 46°40'57.5"N | 15°43'29.6"E | 190,000 | 292 |
| Zahodno Goričko | Pannonian | 46°49'27.6"N | 16°05'09.1"E | 222,000 | 270 |
| Ljutomer | Pannonian | 46°32'09.9"N | 16°11'54.8"E | 214,000 | 175 |
| Murska Sobota | Pannonian | 46°39'29.4"N | 16°09'47.5"E | 218,000 | 189 |
| Radenci | Pannonian | 46°38'35.2"N | 16°02'20.1"E | 209,000 | 202 |
| Ormož | Pannonian | 46°25'10.7"N | 16°08'46.2"E | 203,000 | 218 |
| Vzhodno Goričko | Pannonian | 46°49'57.4"N | 16°17'04.8"E | 235,000 | 242 |
| Gornja Radgona | Pannonian | 46°40'38.7"N | 15°59'23.5"E | 207,000 | 206 |
| Ponikva | Pannonian | 46°15'14.1"N | 15°26'32.4"E | 147,000 | 183 |
| Slovenj Gradec | Pannonian | 46°30'28.0"N | 15°04'37.2"E | 138,000 | 262 |
| Šmarje | Pannonian | 46°14'08.6"N | 15°31'30.9"E | 153,000 | 592 |
| Bistrica | Pannonian | 46°23'20.5"N | 15°34'07.4"E | 127,000 | 201 |
| Bohinj | Alpine | 46°17'36.4"N | 13°54'40.5"E | 64,000 | 308 |
| Kobarid | Alpine | 46°14'52.7"N | 13°34'43.9"E | 53,000 | 235 |
| Tolmin | Alpine | 46°11'08.9"N | 13°43'52.5"E | 48,000 | 600 |
| Kamnik | Alpine | 46°13'01.5"N | 14°37'04.8"E | 91,000 | 340 |
| Ribnica na Pohorju | Alpine | 46°32'09.1"N | 15°16'05.5"E | 153,000 | 276 |
| Mozirje | Alpine | 46°20'21.3"N | 14°57'40.5"E | 121,000 | 449 |
| Savinjska | Alpine | 46°15'57.3"N | 15°07'13.4"E | 127,000 | 338 |
| Vitanje | Alpine | 46°22'48.7"N | 15°17'41.8"E | 144,000 | 322 |
| Prevalje | Alpine | 46°32'56.5"N | 14°55'19.1"E | 134,000 | 413 |
| Vrh | Alpine | 46°34'30.5"N | 15°02'05.0"E | 141,000 | 346 |
| Dravograd | Alpine | 46°35'23.5"N | 15°01'21.8"E | 141,000 | 390 |
| Vuzenica | Alpine | 46°35'53.8"N | 15°09'54.4"E | 150,000 | 365 |
| Polzela | Alpine | 46°16'50.4"N | 15°04'29.9"E | 124,000 | 292 |
| Zavodnje | Alpine | 46°25'29.8"N | 15°01'02.9"E | 129,000 | 642 |
| Škale | Alpine | 46°23'28.1"N | 15°05'29.9"E | 132,000 | 234 |
| Planina | Alpine | 46°06'17.2"N | 15°24'17.4"E | 140,000 | 275 |
| Braslovče | Alpine | 46°17'18.8"N | 15°02'20.8"E | 123,000 | 456 |
| Vinica | Dinaric | 45°27'41.5"N | 15°15'12.6"E | 60,000 | 561 |
| Velike Lašče | Dinaric | 45°50'05.3"N | 14°38'18.6"E | 62,000 | 307 |
| Kamenje | Dinaric | 45°51'08.3"N | 15°08'58.5"E | 82,000 | 184 |
| Logatec | Dinaric | 45°55'01.6"N | 14°13'32.7"E | 48,000 | 526 |
| Žužemberk | Dinaric | 45°49'53.4"N | 14°55'41.3"E | 70,000 | 387 |
| Podpeč | Dinaric | 45°58'22.9"N | 14°24'56.5"E | 62,000 | 476 |
| Postojna | Dinaric | 45°46'29.4"N | 14°12'55.7"E | 39,000 | 209 |
| Brežice | Dinaric | 45°55'17.1"N | 15°35'50.1"E | 149,000 | 292 |
| Sevnica | Dinaric | 46°00'42.5"N | 15°18'32.5"E | 126,000 | 556 |
